# Comparison of Single and Combined Treatment with Exercise Therapy and Collagen Supplementation on Early Knee Arthritis among Athletes—A Quasi-Randomized Trial

**DOI:** 10.3390/ijerph20237088

**Published:** 2023-11-21

**Authors:** Dias Tina Thomas, Ashish John Prabhakar, Charu Eapen, Vivek D. Patel, Vijayakumar Palaniswamy, Molly Cynthia Dsouza, Shruthi R, Yogeesh Dattakumar Kamat

**Affiliations:** 1 Department of Physiotherapy, Kasturba Medical College, Mangalore, Manipal Academy of Higher Education, Manipal, India; tina.mchpmlr2022@learner.manipal.edu (D.T.T.); charu.eapen@manipal.edu (C.E.); vivekdpatel231188@gmail.com (V.D.P.); vijayakumar.kmcmlr@manipal.edu (V.P.); molly.cynthia@manipal.edu (M.C.D.); shruthiravi0097@gmail.com (S.R.); 2 Adjunct Faculty, Department of Orthopaedics, Kasturba Medical College Hospital, Ambedkar Circle, Mangalore, India; yogeesh.kamat@manipal.edu; 3 Consultant Hip and Knee Surgeon, KMC Hospital, Ambedkar Circle, Mangalore, India

**Keywords:** early onset arthritis, knee, exercises, rehabilitation, collagen, muscle strength, exercise therapy

## Abstract

Athletic injuries are commonly implicated in the development of early osteoarthritic (EOA) changes in the knee. These changes have a significant impact on athletic performance, and therefore the early detection of EOA is paramount. The objective of the study is to assess the impact of different interventions on individuals with EOA, particularly focusing on recreational athletes. The study aims to evaluate the effectiveness of three treatment groups in improving various aspects related to knee EOA, including pain, range of motion, strength, and function. A study was undertaken with 48 recreational athletes with EOA who were assigned to one of three groups by the referring orthopedic surgeon: collagen (Col), exercise (Ex), or collagen and exercise (ColEx) groups. All the participants received their respective group-based intervention for 12 weeks. Visual analog scale (VAS), knee flexion range of motion (ROM) knee flexors and extensors strength, and KOOS were assessed at baseline, and after 4 weeks, 8 weeks, and 12 weeks of intervention. VAS for activity improved in all treatment groups, with no difference between groups. The between-group analysis for knee ROM revealed a significant difference (*p =* 0.022) in the Col vs. Ex group at 12 weeks. The knee flexor and extensor strength and the KOOS scores improved considerably in the Ex and the ColEx group (*p* < 0.05) at 12 weeks. Exercise therapy improved pain, strength and function in subjects with EOA, whereas the association of collagen seems to have accentuated the effects of exercise in bringing about clinical improvements.

## 1. Introduction

Sports are physical activities that require skill. Sporting activities and their capacity to cause musculoskeletal problems have traditionally been the subject of much discussion [1]. Athletes in competitive sports must engage in more intensive and strenuous training programs, as well as more practice and playing hours [2]. The mean athletic age group is between 14 years and 52.8 years, wherein 99% of the athletes are aged below 40 years, and 72% of the athletes are aged between 20 and 30 years [3]. The prevalence of knee discomfort leading to early arthritic changes among soccer players is ascribed to previous knee injuries and has a prevalence of 16–80% worldwide in the 30–35-years age group [4].

The knee joint is a functional unit that is responsible for transmitting load during both extensive and daily activities. The magnitude of the load can range from exceedingly low to very large. Sporting activities place a large level of repetitive stress on the tibiofemoral joint. Hence, it becomes imperative to detect these arthritic changes within the knee joint early and introduce primary interventions and therapeutic approaches that could prevent further progression of the disease [5].

Osteoarthritis (OA) is a global health problem that affects over 300 million people and is a major cause of disability. In India, the prevalence of knee OA is found to be 28.7% and approximately 80% of Indians who claim to have knee pain show signs of OA, out of which 20% report an incapability to carry out daily activities [6]. 

Knee OA is important not only due to its high prevalence rate compared to other types of OA, but because it is increasingly being reported in the young and in athletic populations [7]. The prevalence of knee discomfort leading to early arthritic changes among soccer players is ascribed to previous knee injuries and has a prevalence of 16–80% worldwide in the 30–35-years age group [4]. Considering this, it has been suggested that the early detection and primary prevention of knee OA should become a main aim of health care.

The identification of risk factors in EOA is crucial to initiate adequate and prompt conservative management and to prevent progression of the condition to the point where surgical intervention becomes necessary [8].

Etiological considerations contributing to EOA can be broadly classified into endogenous and exogenous factors. Genetic factors, especially those coding for the structural proteins in type II collagen, gender, where studies show that females are more prone to developing osteoarthritis because of the anatomical difference that exists between the genders, various ethnic groups, and the age of the individual are the endogenous factors contributing to the disease [9,10].

External factors like meniscal tears, which may accelerate the process of arthritic changes, obesity, i.e., a BMI > 30, injuries involving the ligaments of the knee, which may lead to decreased joint stability and could cause joint degeneration, and acquired knee axis deviations and various nutritional deficits, are a few factors that could account for the early onset of the disease [9,11].

The first line of management for knee pain associated with arthritic changes is often pharmacological, followed by therapeutic intervention [12]. Acetaminophen was consistently advocated as the first-line drug, followed by topical applications like capsaicin and NSAIDS for pain management [9,13]. Non-pharmacological approaches to the treatment of osteoarthritis (OA) have evolved to include biologic interventions, expanding the treatment landscape. Biologic therapies encompass the use of autologous treatments such as platelet-rich plasma (PRP), stem cells, or a combination of both. These innovative approaches aim to harness the regenerative potential of the body’s own biological components, offering promising avenues for OA management by promoting tissue repair and mitigating symptoms [14]. 

A 12-week program of collagen supplementation is known to significantly reduce self-reported pain in young athletes [15]. Results from experimental studies demonstrate that collagen can alleviate articular pain, as well as levels of cross-linked C-telopeptide of type II collagen (CTX-II), a biomarker of cartilage degradation [16]. Furthermore, the stimulation of cultured chondrocytes by hydrolysed collagen results in the production of type II collagen and proteoglycans, which help to reduce pain and improve function [17].

Management of knee OA can be broadly categorized as pharmacological and non-pharmacological. Therapeutic interventions are targeted to reduce pain, increase strength, and improve knee joint function. Exercise programs implemented for a period of 16 weeks are known to improve muscle strength, reduce pain, and improve the functionality of the knee joint [18]. Combined kinetic exercises [19] and routine therapeutic exercises focus on improving flexibility, strength, range of motion, and endurance. Exercise therapy may be more advantageous for individuals who are younger, have knee osteoarthritis (OA), and are not currently in the process of preparing for joint replacement surgery [20].

An agent with a reported positive effect on self-reported knee pain in athletes is hydrolyzed type I and native type II collagen. A 12-week program of collagen supplementation is known to significantly reduce self-reported pain in young athletes [15]. Results from experimental studies demonstrate that collagen can alleviate articular pain as well as levels of cross-linked C-telopeptide of type II collagen (CTX-II), a biomarker of cartilage degradation [16]. Furthermore, the stimulation of cultured chondrocytes via hydrolyzed collagen results in the production of type II collagen and proteoglycans, which thereby help to reduce pain and improve function [17]. Collagen-based supplements are effective in alleviating OA symptoms, as evidenced by improvements in the total WOMAC index and VAS scores [21].

In light of the aforementioned considerations, the present study aims to investigate the effectiveness, superiority, and relative benefits of three distinct treatment paradigms for athletes diagnosed with early arthritic changes in the knee. Specifically, the study will evaluate the impact of collagen supplementation, exercise, and exercise along with collagen supplementation, as modes of intervention, on alleviating knee pain, improving joint function, and potentially slowing the progression of knee OA. Hence, against the above background, the present study is undertaken to understand the effectiveness and superiority, and to review the relative benefits, of three different treatment paradigms in athletes diagnosed with early arthritic changes in the knee.

## 2. Methods

### 2.1. Design and Participants 

A double-blind, quasi-randomized trial was conducted in the hospital settings of Kasturba Medical college, Mangalore, between March 2021 and March 2022. The primary outcomes were measured using the pain scale and assessed using the visual analogue scale (VAS). The functional outcomes were measured using the knee injury and osteoarthritis outcome score (KOOS) and the secondary outcomes were knee muscle strength and knee muscle range of motion (ROM). A total sample size of 48 was included and a standard deviation of 0.74 was acquired from a previous study at a confidence level of 95% and precision d = 0.86 [19].

**Figure 1:**
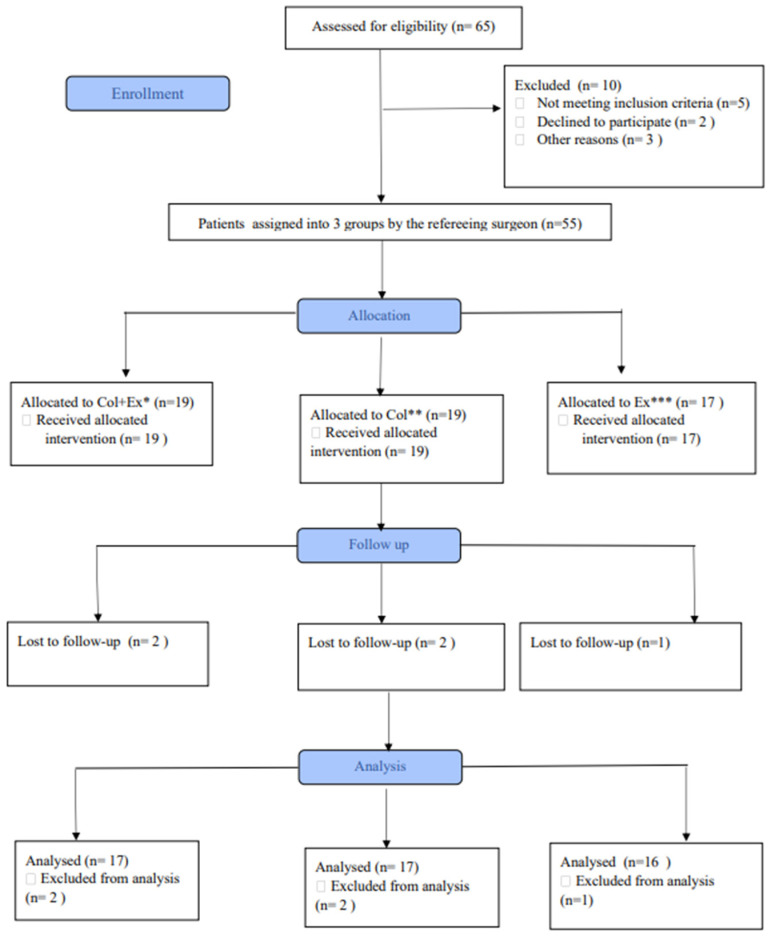
Patient recruitment and study design flowchart. * collagen + exercise ** collagen *** exercise.

Participants were randomly allocated by the referring surgeon using a random number table, and subjects were randomly allocated using the non-purposive sampling technique in one of the routinely followed and prescribed treatment groups, i.e., group (1) was the exercise therapy group, group (2) the collagen supplementation group, and group (3) underwent a combined intervention of exercise therapy and collagen supplementation (Figure 1).

The inclusion criteria were as follows: (a) athletes above the age of 18 and below 55; (b) knee pain and stiffness that interferes with sports performance; (c) grades I and II on the Kellgren Lawrence scale, as diagnosed and confirmed by an orthopedician. The participants were screened based on the study’s eligibility requirements. The participants were excluded if they had any recent trauma or injury to the knee or any surgery in the past 3 months. 

### 2.2. Instruments 

A handheld dynamometer was used to assess the muscle strength, ROM was assessed using the universal goniometer, and the KOOS scale was used to evaluate the patient-reported outcomes. 

### 2.3. Intervention

Group 1—exercise therapy group;

Group 2—collagen supplementation;

Group 3—combined intervention of exercise therapy and collagen supplementation.

The exercises prescribed to both groups were:

(1) hip muscle strengthening exercises, including hip flexors, abductors, adductors, and extensors;
(2) knee muscle strengthening exercises, including dynamic quadriceps exercise, hamstring curls, gastrocnemius stretching, and squatting;
(3) cycling.

### 2.4. Data Collection

The study received ethical approval from the Institutional Ethics Committee, KMC Mangalore, Manipal Academy of Higher Education (IECKMC MLR 02-2021/54). The study was enrolled into India’s clinical trial registry under the identifier CTRI/2021/04/033091. 

Recreational athletes of either gender, aged from 18 to 52 years, with no current history of musculoskeletal injury, were recruited after providing their written informed consent. Demographic data and baseline data were noted on the day the patient was referred for physiotherapy. The data included a 10 cm visual analogue scale (VAS), the knee injury and osteoarthritis outcome score (KOOS), knee flexion range of motion (ROM), which was measured using a goniometer, and knee flexor and extensor muscle strength, which was quantified using a handheld dynamometer. All of the aforementioned outcome measures were assessed at 4 weeks, 8 weeks, and 12 weeks, respectively. A qualified physiotherapist delivered the exercise programs, following which the clinical endpoints were measured by the primary investigator, who was blinded to the study.

### 2.5. Statistical Analysis

The collected data were entered into the Statistical Package for Social Sciences (SPSS) version 21.0. A descriptive analysis of the baseline characteristics of the study subjects was carried out. Baseline data were compared between groups, and as the data were normally distributed, an unpaired *t*-test was used. The between-group comparison was made using repeated-measures ANOVA. Bonferroni post hoc analysis was used to correct the differences between groups. A *p*-value of <0.05 was considered statistically significant. 

## 3. Results

### 3.1. Patient Demographics 

Patient demographics: The study included 48 participants with a mean age of approximately 29–30 years, divided into three groups: collagen + exercise (ColEx), collagen (Col), and exercise (Ex).

### 3.2. Pain Score at Weeks 4, 8 and 12 

Pain score: Pain levels significantly reduced in all three groups over 12 weeks (At rest, ColEx: *p* < 0.001, Col: *p* < 0.001, Ex: *p* < 0.001. During activity, ColEx: *p* < 0.001, Col: *p* < 0.001, Ex: *p* < 0.001. No significant differences were found between the groups.

### 3.3. Knee Flexion ROM at Weeks 4, 8 and 12 

**Figure 2:**
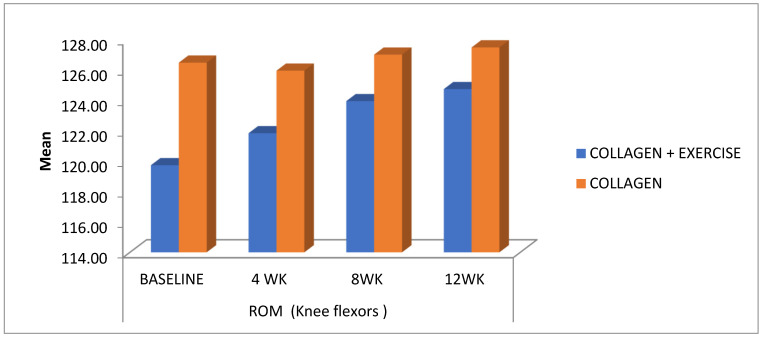
Changes in the range of motion (ROM) of knee flexion among the three treatment groups (collagen + exercise—ColEx, collagen—Col, and exercise—Ex) at different time intervals (4 weeks, 8 weeks, and 12 weeks) during the study period.

The knee flexion ROM (Figure 2) in the ColEx group at baseline was 119.74 ± 10.862, which showed a statistically significant increment to 124.74 ± 7.355 by 12 weeks (*p =* 0.000), whereas the ROM in the Ex-group at baseline was 122.50 ± 9.129, which increased to 129.69 ± 5.313 by 12 weeks (*p* = 0.000). The baseline values for knee ROM in the Col group were 126.47 ± 5.491, but did not show a statistically significant improvement by 12 weeks 127.47 ± 5.197 (*p* = 0.111) (Table 1).

### 3.4. Knee Flexor Strength at Weeks 4, 8 and 12 

The knee flexor strength at baseline was statistically significant in all three groups from baseline to 12 weeks (Table 2). The between-group analysis is shown in (Table 3). Knee flexor and extensor strength significantly improved in all three groups (*p* < 0.001). No significant differences were observed in knee extensor strength between ColEx and Ex (*p* = 1.000), but there was a significant difference between ColEx and Col (*p* < 0.001) and Col vs. Ex (*p* < 0.001).

### 3.5. KOOS Score at Weeks 4, 8 and 12 

KOOS Score: (Table 3) For various components of the knee osteoarthritis outcome scores (KOOS), there were no significant differences between the ColEx and Ex groups (*p* = 1.000). However, significant differences were observed between the ColEx vs. Col group and the Col vs. Ex group in several KOOS components, indicating variations in treatment effects.

All three groups exhibited significant reductions in pain levels over the 12-week study period. The ColEx group experienced a substantial reduction in pain at rest, with a *p*-value of <0.001 when compared to baseline. Pain during activity also significantly improved in all groups, with a *p*-value of <0.001, and there were no statistically significant differences between the groups. Knee flexion range of motion (ROM): knee flexion ROM significantly improved in the ColEx and Ex groups, with *p*-values of <0.001. In contrast, the Col group did not show statistically significant improvements in knee flexion ROM, with a *p*-value of 0.111. Between-group analysis revealed a statistically significant difference in knee ROM improvement between the Col group and Ex group, with a *p*-value of 0.022. Knee strength: Knee flexor strength showed statistically significant improvements in all groups, with *p*-values of <0.001. Knee extensor strength improved significantly in the ColEx, Col, and Ex groups, with *p*-values of <0.001. Knee osteoarthritis outcome scores (KOOS): For various components of the KOOS (ADL, pain, QOL, sports/rec, symptom), there were no statistically significant differences between the ColEx and Ex groups, with *p*-values of 1.000. However, statistically significant differences were observed between the ColEx vs. Col group and the Col vs. Ex group in several KOOS components, indicating variations in treatment effects.

## 4. Discussion

The objective of this study was to determine the efficacy, superiority, and relative benefits of three different paradigms of treatment, i.e., exercise therapy, collagen supplementation, and a combination of collage supplementation with exercise therapy, in alleviating pain, and improving muscle strength, range of motion, and functional scores in athletes diagnosed with EOA.

**Figure 3:**
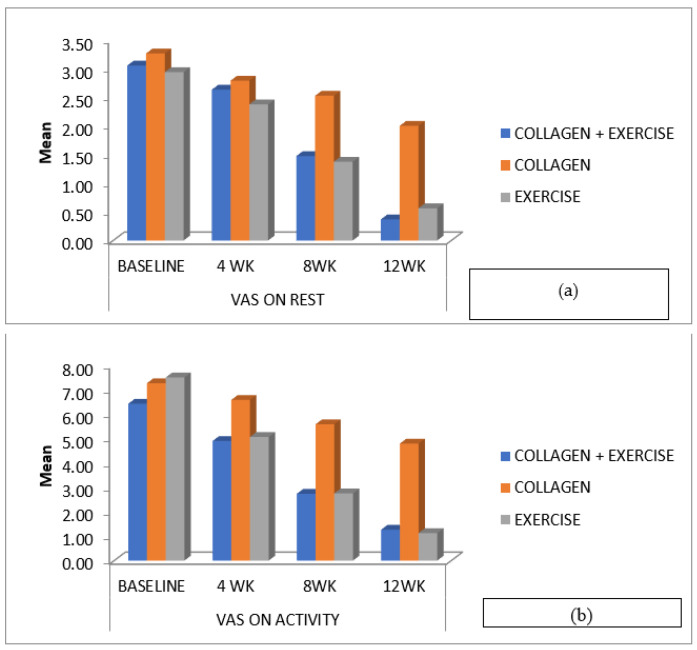
(**a**,**b**) provide a graphical representation of the changes in visual analog scale (VAS) scores among the three treatment groups (collagen + exercise—ColEx, collagen—Col, and exercise—Ex) at various time points (4 weeks, 8 weeks, and 12 weeks). These figures illustrate the differences in pain levels experienced by participants both at rest (**a**) and during activity (**b**) throughout the study period.

VAS on activity (Figure 3b) was significantly improved in all three groups, whereas there was no significant difference in VAS on rest (Figure 3a). The combined administration of collagen and exercise therapy demonstrated greater efficacy in pain reduction compared to the individual administration of collagen or exercise therapy. Pain is the primary concern in EOA, since it precedes other unfavourable aspects of an individual’s life, such as physical inactivity, which contributes to decreasing muscle strength and gait abnormalities [22]. It is critical to recognize that there is an inflammatory signalling pathway that leads to pain and muscular weakness.

In our study, athletes diagnosed with EOA were managed conservatively using either exercise therapy (routine exercises + proprioceptive exercises), oral collagen supplements, or both. The implications for exercise and pain reduction in an arthritic joint include Interleukin 1 beta [23], which stimulates the synthesis of prostaglandins and nitrous oxide, which leads to reduced proteoglycan formation and ECM. According to Chowdhury and colleagues, the dynamic compression of chondrocytes suppresses the release of prostaglandins and nitrous oxide. As a result, dynamic mechanical compression of the arthritic knee joint may aid in deteriorating the inflammatory process. This compression could be duplicated during therapeutic exercises by exercises that place a dynamic and physiological load on the knee joint [23]. Another key aspect that contributes to pain relief after exercise is the facilitation of endorphin release, which makes the patient more tolerant of pain [24].

The findings of our study are in line with another 12-week study of participants with knee OA, which concluded that strengthening and coordination exercises conducted three times a week reduced pain in 63% of sessions [25].

In the present study, it was also observed that the collagen group did not demonstrate a statistically significant difference in pain relief when compared to the other groups (Table 1). Previous research, however, has indicated that collagen supplementation has a favourable effect on pain when taken over 24 weeks [26,27,28]. In our study, collagen supplementation was administered for 12 weeks, thus implying that long-term supplementation may be required to provide beneficial effects on knee pain.

Differences in joint mobility were measured using the ROM method and isometric muscle strength was quantified with a hand-held dynamometer. We observed a moderate to strong improvement in the ROM and knee muscle strength in individuals with EOA in the Ex-group. The ROM of the arthritic knee joint is thought to be a crucial determinant in the development of muscle weakness during isokinetic activity [5]. Patients’ symptoms may be exacerbated by restricted soft tissue mobility and adhesions caused by prolonged inflammation of both intra- and periarticular tissues [29].

**Figure 4:**
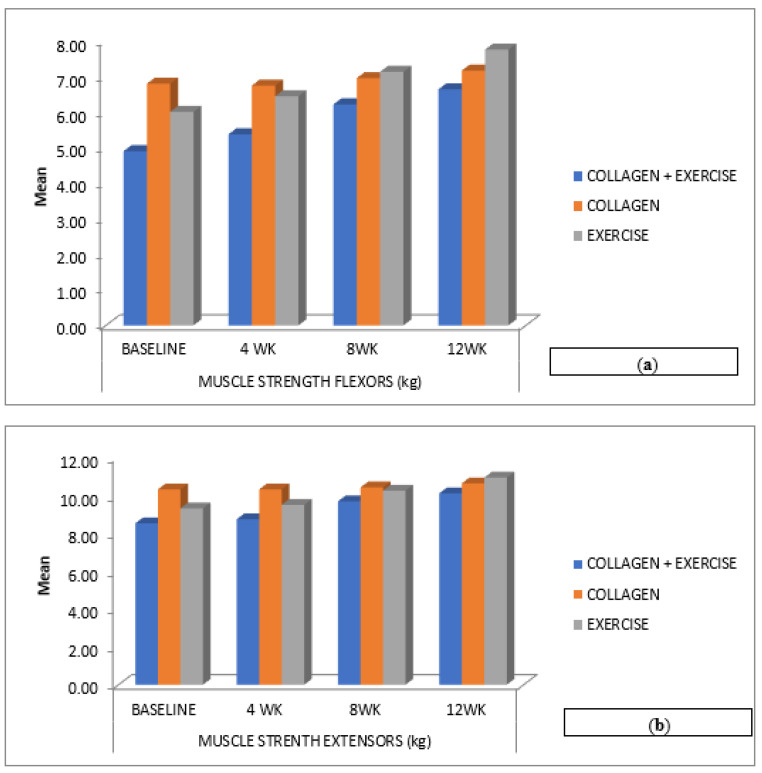
(**a**,**b**) Changes in knee muscle strength within the three treatment groups (collagen + exercise—ColEx, collagen—Col, and exercise—Ex) at different time points (4 weeks, 8 weeks, and 12 weeks) throughout the study.

In the present study, active knee flexion ROM increased significantly in the Ex-group as compared to the other groups (Figure 2), whereas, on the other hand, there was a noticeable improvement in the knee flexor and extensor strength in the ColEx and Ex groups. However, the group that underwent administered exercises showed a greater quadriceps strength than the other intervention groups (Figure 4a,b). Pain relief following strengthening activities can be ascribed to the reversal of quadriceps’ weakness, which is common in EOA. Strength training can increase muscle strength and reduce pain at the same time [5].

In our present study, the KOOS scores showed a statistically significant difference for the ColEx and the Ex-group at the end of 12 weeks (Table 2). A plausible explanation for, the improvement in the functional scores in the ColEx and Ex group can be attributed to improvements in the pain scores, muscle strength, and knee ROM. Stronger muscles allow for a person to undertake physical activities like walking and climbing stairs more effortlessly. Such improvements in their functional activities and daily performance can contribute to a sense of self-efficacy and reduce activity avoidance.

EOA is a constellation of anatomical abnormalities that cause functional disability and pain. There are various risk factors associated with disease progression; however, it appears that early joint damage or trauma during sporting events is a more common cause in the athletic population. The assessment and effective management of EOA in an athlete is frequently difficult due to his or her increased exercise demands and requirement for an accelerated return to sport. Athletes who participate in sports that require quick acceleration and instant deceleration, who engage in continuous training with an impact on joints, or who compete at an elite level for extended periods are at a higher risk of developing EOA [1].

EOA management should always include a variety of therapeutic options targeted at alleviating pain and improving function. The recommended management framework should prioritize nonpharmacological techniques, followed by nutritional supplements and analgesics. In the present study, the ColEx group displayed significant changes in all the investigated parameters; however, exercise therapy is a standalone treatment.

Considerably significant changes were seen in the results when compared to individuals treated with collagen supplementation alone. Therefore, the authors highly recommend using oral collagen supplementation along with exercise as the treatment of choice in EOA.

We accept that our study had several limitations. As subject randomization was achieved through allocation and not concealment, a possible selection bias could have occurred. Second, because this study was conducted on recreational athletes, the capacity to extend these findings to a larger population is limited, and lastly, the sample size within the included groups is relatively small.

## 5. Conclusions and Future Directions

The influence of sports practice on the development of EOA is a cause for concern. The ability to treat OA in its early stages may be largely linked to alterations in specific extrinsic or intrinsic factors associated with the patient to spare the joint from further disease progression; these factors include sports practice, equipment, and load. Nonpharmacological management such as muscle strengthening, and training plays an important role in the care of athletes with EOA. The present study concluded that exercise therapy as a standalone treatment exhibited considerably significant changes; however, exercise therapy combined with oral collagen supplementation was effective and superior to the other interventions in improving the VAS scores, strength, and KOOS scores in people with EOA. Further studies need to be conducted, wherein the effect of exercise and collagen supplementation on cartilage health should be correlated with the radiological findings and clinical biomarkers for EOA.

## Figures and Tables

**Table 1:** Within-group difference comparison of pain outcomes using the VAS scale and KOOS—functional outcomes among the three groups over time, i.e., baseline, 4 weeks, 8 weeks, and 12 weeks, along with corresponding *p*-values to indicate statistical significance. * A *p*-value of <0.05 is considered statistically significant. * *p* < 0.05, ** *p* < 0.01, *** *p* < 0.001, **** *p* < 0.0001, wherein the *p* values are compared to the baseline score.

Outcome	Time	ColEx	Col	Ex	*p*-Value ColEx	*p*-Value Col	*p*-Value Ex
		Mean ± SD	Mean ± SD	Mean ± SD	(*p* < 0.05)	(*p* < 0.05)	(*p* < 0.05)
VAS on rest	Baseline	3.05 ± 1.649	3.26 ± 1.628	2.94 ± 1.769			
	4 WK	2.63 ± 1.802	2.79 ± 1.437	2.38 ± 1.544	0.119	0.008 **	0.007 **
	8 WK	1.47 ± 1.349	2.53 ± 1.504	1.38 ± 1.204	0.0001 ****	0.0001 ****	0.0001 ****
	12 WK	0.37 ± 0.597	2.00 ± 1.291	0.56 ± 0.629	0.0001 ****	0.0001 ****	0.0001 ****
VAS on activity	Baseline	6.42 ± 1.216	7.26 ± 1.327	7.50 ± 0.894			
	4 WK	4.89 ± 1.883	6.58 ± 1.305	5.06 ± 0.998	0.0001 ****	0.001 ***	0.0001 ****
	8 WK	2.74 ± 1.327	5.58 ± 1.387	2.75 ± 1.125	0.0001 ****	0.0001 ****	0.0001 ****
	12 WK	1.26 ± 0.872	4.79 ± 1.548	1.13 ± 1.769	0.0001 ****	0.0001 ****	0.0001 ****
KOOS ADL	Baseline	50.74 ± 12.823	46.84 ± 17.069	54.88 ± 8.801			
	4 WK	60.42 ± 9.063	51.00 ± 13.178	68.75 ± 7.479	0.0001 ****	0.002 *	0.0001 ****
	8 WK	75.47 ± 6.883	54.84 ± 11.899	82.38 ± 8.065	0.0001 ****	0.0001 ****	0.0001 ****
	12 WK	87.32 ± 6.709	59.05 ± 12.236	89.25 ± 5.040	0.0001 ****	0.0001 ****	0.0001 ****
KOOS pain	Baseline	48.11 ± 13.984	43.68 ± 12.845	50.19 ± 9.897			
	4 WK	59.26 ± 10.514	46.05 ± 12.058	66.50 ± 10.040	0.001 ***	0.038 *	0.0001 ****
	8 WK	74.26 ± 7.164	51.00 ± 9.383	80.63 ± 9.323	0.0001 ****	0.001 ***	0.0001 ****
	12 WK	85.26 ± 6.707	57.84 ± 11.969	89.50 ± 40761	0.0001 ****	0.0001 ****	0.0001 ****
KOOS QOL	Baseline	36.00 ± 11.324	35.63 ± 15.582	39.56 ± 10.013			
	4 WK	47.42 ± 8.965	42.89 ± 13.008	56.38 ± 10.443	0.0001 ****	0.003 *	0.0001 ****
	8 WK	65.26 ± 7.362	50.47 ± 12.330	69.94 ± 9.849	0.0001 ****	0.0001 ****	0.0001 ****
	12 WK	75.74 ± 69.10	55.05 ± 9.936	77.50 ± 8.532	0.0001 ****	0.0001 ****	0.0001 ****
KOOS sports/rec	Baseline	36.05 ± 17.206	26.05 ± 14.296	37.56 ± 9.919			
	4 WK	50.53 ± 13.218	33.68 ± 13.107	57.63 ± 10.782	0.0001 ****	0.003 ****	0.0001 ****
	8 WK	68.68 ± 9.405	44.21 ± 9.897	74.56 ± 8.148	0.0001 ****	0.0001 ****	0.0001 ****
	12 WK	79.47 ± 8.481	50.26 ± 7.723	82.76 ± 7.767	0.0001 ****	0.0001 ****	0.0001 ****
KOOS symptoms	Baseline	54.16 ± 18.679	44.21 ± 10.438	60.25 ± 15.864			0.0001 ****
	4 WK	61.11 ± 12.635	46.16 ± 9.518	74.25 ± 9.657	0.037 *	0.057 *	0.0001 ****
	8 WK	76.16 ± 9.002	54.79 ± 7.850	85.56 ± 7.248	0.0001 ****	0.0001 ****	0.0001 ****
	12 WK	86.37 ± 8.050	59.00 ± 9.292	90.81 ± 5.294	0.0001 ****	0.0001 ****	0.0001 ****

**Table 2:** Within-group difference comparison of outcomes of knee flexion ROM and muscle strength of knee flexors and extensors among the three groups over time, i.e., baseline, 4 weeks, 8 weeks, and 12 weeks, along with corresponding *p*-values to indicate statistical significance. * A *p*-value of <0.05 is considered statistically significant.

Outcome	Time	ColEx	Col	Ex	*p*-Value ColEx	*p*-Value Col	*p*-Value Ex
ROM (knee flexors)	Baseline	119.74 ± 10.862	126.47 ± 5.491	122.50 ± 9.129			
	4 WK	121.84 ± 8.852	125.95 ± 6.527	126.50 ± 5.888	0.016 *	0.331	0.081
	8 WK	123.95 ± 7.920	127.00 ± 5.568	129.69 ± 5.313	0.001 *	0.163	0.012 *
	12 WK	124.74 ± 7.355	127.47 ± 5.197	129.69 ± 5.313	0.001 *	0.111	0.012 *
Muscle strength (flexors)	Baseline	4.89 ± 1.370	6.79 ± 3.084	6.00 ± 2.366			
	4 WK	5.37 ± 1.422	6.74 ± 3.160	6.44 ± 2.529	0.003 *	0.331	0.110
	8 WK	6.21 ± 1.475	6.95 ± 3.135	7.13 ± 2.527	0.000 *	0.268	0.000 *
	12 WK	6.63 ± 1.383	7.16 ± 3.351	7.75 ± 2.408	0.000 *	0.031 *	0.000 *
Muscle strength (extensors)	Baseline	8.53 ± 2.736	10.32 ± 3.351	9.31 ± 3.439			
	4 WK	8.74 ± 2.746	10.32 ± 3.318	9.50 ± 3.307	0.259	1.000	0.270
	8 WK	9.68 ± 2.849	10.42 ± 3.237	10.25 ± 3.568	0.000 *	0.494	0.000 *
	12 WK	10.11 ± 2.622	10.63 ± 3.270	10.94 ± 3.376	0.000 *	0.111	0.000 *

**Table 3:** Analyses of the between-group analysis.

	TIME	ColEx vs. Col (*p* < 0.05)	ColEx vs. Ex (*p* < 0.05)	Col vs. Ex (*p* < 0.05)
VAS on rest	Baseline - 4 WK	1.000	1.000	1.000
	Baseline - 8 WK	0.081	1.000	0.113
	Baseline - 12 WK	0.010 *	1.000	0.077
VAS on activity	Baseline - 4 WK	0.083	0.069	0.000 *
	Baseline - 8 WK	0.000 *	0.041 *	0.000 *
	Baseline - 12 WK	0.000 *	0.023 *	0.000 *
ROM (knee flexion)	Baseline - 4 WK	0.388	0.880	0.043 *
	Baseline - 8 WK	0.218	0.488	0.008 *
	Baseline - 12 WK	0.193	0.983	0.022 *
Muscle strength (flexors)	Baseline - 4 WK	0.063	1.000	0.116
	Baseline - 8 WK	0.000 *	1.000	0.000 *
	Baseline - 12 WK	0.000 *	1.000	0.000 *
Muscle strength (extensors)	Baseline - 4 WK	0.900	1.000	1.000
	Baseline - 8 WK	0.000 *	1.000	0.002 *
	Baseline - 12 WK	0.000 *	1.000	0.000 *
KOOS ADL	Baseline - 4 WK	0.069	0.286	0.001 *
	Baseline - 8 WK	0.000 *	1.000	0.000 *
	Baseline - 12 WK	0.000 *	1.000	0.000 *
KOOS pain	Baseline - 4 WK	0.017 *	0.337	0.000 *
	Baseline - 8 WK	0.000 *	0.727	0.000 *
	Baseline - 12 WK	0.000 *	1.000	0.000 *
KOOS QOL	Baseline - 4 WK	0.517	0.277	0.011 *
	Baseline - 8 WK	0.000 *	1.000	0.000 *
	Baseline - 12 WK	0.000 *	1.000	0.000 *
KOOS sports/rec	Baseline - 4 WK	0.142	0.355	0.003 *
	Baseline - 8 WK	0.001 *	0.865	0.000 *
	Baseline - 12 WK	0.000 *	1.000	0.000 *
KOOS symptoms	Baseline - 4 WK	0.379	0.123	0.002 *
	Baseline - 8 WK	0.008 *	1.000	0.001 *
	Baseline - 12 WK	0.000 *	1.000	0.002 *

* *p*-Values less than 0.05 (*) indicate statistically significant differences. *p* values are compared with the baseline. ColEx: collagen supplementation + exercise group, Col: collagen supplementation only group, Ex: exercise only group, VAS: visual analog scale, ROM: range of motion, KOOS: knee injury and osteoarthritis outcome score, ADL: activities of daily living: quality of life, sports/rec: sport and recreation, symptoms: symptom assessment.

## Data Availability

Available on OSF–DOI 10.17605/OSF.IO/V6Y9P.
